# Profiling miRNA in salivary samples from subjects with endometriosis: a pilot study

**DOI:** 10.3389/fmolb.2026.1869937

**Published:** 2026-07-22

**Authors:** Graziella Calugi, Francesca Blandino, Carla Proserpio, Luca Forlani, Noemi Meschino, Riccardo Giannico, Fabio Dalla Pozza, Andrea Graziani, Lucia Di Pietro, Lucia Buccarello, Francesco Giuseppe Martire, Davide Dealberti, Gabriele Lanzo, Stefano Cosma, Gianluigi Marchino, Stefano Luisi, Gianluca Raffaello Damiani, Elisabetta Garavaglia, Stefano Landi, Massimiliano Marziali, Annalisa Liprino, Valentina Violante Pontello, Stefano Fracchioli, Giuseppe Caruso

**Affiliations:** 1 Eurofins Genoma Group, Research and Development Department, Rome, Italy; 2 Eurofins Genoma Group, Bioinformatic Department, Rome, Italy; 3 Department of Pharmaceutical Chemistry, The University of Kansas, Lawrence, KS, United States; 4 Department of Biomedical and Biotechnological Sciences, University of Catania, Catania, Italy; 5 Departmental Faculty of Medicine, UniCamillus—Saint Camillus International University of Health Sciences, Rome, Italy; 6 IRCCS San Camillo Hospital, Venice, Italy; 7 UOC Gynecology Department of Molecular and Development Medicine, University of Siena, Siena, Italy; 8 Biagio and Arrigo, Obstetrics and Gynecology, University Hospital Company SS Antonio, Alessandria, Italy; 9 Department of Health and Surgical Science, Gynecology and Obstetrics, S. Anna Hospital, University of Turin, Turin, Italy; 10 GVM S. Catherine of Siena Clinic, Turin, Italy; 11 Obstetrics and Gynecology, AOUP Pisana Universiy-Hospital Company, Pisa, Italy; 12 Policlinic of Bari, Obstetrics and Gynecology, UOC University-Hospital Company, Bari, Italy; 13 Department of Obstetrics and Gynecology, Sesto S. Giovanni Hospital, Milan, Italy; 14 Department of Obstetrics and Gynecology, Moriggia Pelascini-Gravedona General Hospital, Como, Italy; 15 Department of Obstetrics and Gynecology, New Villa Claudia Clinic, Rome, Italy; 16 UMR Unit of Reproduction Medicine, Hera Center, Catania, Italy; 17 Pontello Gynecology Clinic, Florence, Italy; 18 Fracchioli Gynecology Clinic, Turin, Italy

**Keywords:** endometriosis, inflammation, miRNA, non-invasive biomarkers, oxidative stress, saliva

## Abstract

**Introduction:**

Endometriosis is a common, chronic, estrogen-dependent gynecological disorder characterized by the growth of endometrial-like tissue outside the uterus and frequently associated with pelvic pain and infertility. Despite its high prevalence, the molecular mechanisms underlying lesion persistence and inflammation remain poorly understood, limiting the development of reliable non-surgical diagnostic tools and targeted therapies.

**Methods:**

A case-control study included 176 women with endometriosis and 124 controls. Salivary microRNA (miRNA) expression was analyzed using next-generation sequencing. Patients were stratified into untreated and treated groups (pharmacological or surgical therapy), while controls included healthy individuals and a technical control group of women with benign gynecological conditions to reduce potential confounding factors.

**Results:**

Ten salivary miRNAs were associated with disease activity. Seven (hsa-miR-130a-3p, hsa-miR-130b-3p, hsa-miR-141-3p, hsa-miR-200b-3p, hsa-miR-200c-3p, hsa-miR-203b-5p, and hsa-miR-29c-3p) had previously been linked to endometriosis, while three were associated with inflammatory or cancer-related pathways. Expression levels showed a gradient across clinical groups, with intermediate levels in treated patients, suggesting therapeutic modulation. Target analysis identified PTEN as a key regulated gene, implicating the PI3K/AKT/mTOR pathway.

**Discussion:**

These findings support salivary miRNAs as promising non-invasive biomarkers for the diagnosis and monitoring of endometriosis and provide further insight into the molecular pathways involved in disease pathogenesis.

## 1 Introduction

Endometriosis is a common, chronic, and estrogen-dependent gynecological condition defined by the presence of endometrial-like tissue (glands and stroma) in ectopic locations (Alimi et al., 2018). The lesions are primarily found in the pelvic peritoneum, ovaries, and rectovaginal septum (Alimi et al., 2018). In light of these widespread effects, endometriosis should be considered a public health issue rather than merely a disease of individuals. Endometriosis can be categorized into three well-recognized phenotypes: superficial peritoneal lesions (SUP), ovarian endometriomas (OMA), and deep infiltrating endometriosis (DIE). OMA are cystic masses derived from ectopic endometrial tissue within the ovary, while the most severe phenotype, DIE, is defined as sub-peritoneal lesions that penetrate tissue deeper than 5 mm under the peritoneal surface, potentially infiltrating organs surrounding the uterus, such as bladder or intestine (Chapron et al., 2010; Chapron et al., 2003), leading to its characterization as an “abdominal-pelvic multifocal disease” (Chapron et al., 2010). Furthermore, the presence of OMA is often an indicator of more severe associated DIE (Anonymous, 1985; Anonymous, 1997; Leyend et al., 2015) and independently contributes to pain (Vercellini et al., 2014a; Naftalin et al., 2014). The disease is characterized by a wide variability in presentation and symptoms, which include dysmenorrhea, dyspareunia, chronic pelvic pain, irregular uterine bleeding, and/or infertility (Bonavina and Taylor, 2022). Notably, infertility affects 30%–50% of women diagnosed with endometriosis (Bonavina and Taylor, 2022). The most well-accepted pathophysiological hypothesis for endometriosis is based on retrograde menstruation, where menses transport viable endometrial fragments through the fallopian tubes to the peritoneal cavity for implantation (Bricou et al., 2008; Vercellini et al., 2014b). Genetically, endometriosis is considered a complex trait that exhibits familial aggregation, with up to a six-fold increased risk for first-degree relatives of patients (Stefansson et al., 2002; Treloa et al., 1999; Rahmioglu et al., 2012; Treloar et al., 2002), underscoring the complex, multi-factorial nature of the disease. The natural course of endometriosis is characterized by cyclic bleeding (Menni et al., 2016) and repeated tissue injury and repair at the site of the ectopic lesions, resulting in local inflammation (Laux-Biehlmann et al., 2015; McKinnon et al., 2015; Zhang et al., 2016). Endometriosis-related pain is mediated by several mechanisms. Ectopic endometrial cells outside the uterus stimulate immune cell infiltration, which secretes inflammatory mediators (such as cytokines and nerve growth factor), fostering a proinflammatory peritoneal microenvironment (McKinnon et al., 2015). The close topographical relationship between endometriotic foci and nerves (Anaf et al., 2006; Anaf et al., 2000; Hoffman, 2015) as well as structural changes within the central pain system (Anaf et al., 2000; As-Sanie et al., 2012; Khan et al., 2013; Zhang et al., 2010) all contribute to pelvic pain (Chapron et al., 2012). Endometriosis-related infertility is a clear association, although the diagnosis is not synonymous with infertility. Adverse effects on fecundity include chronic inflammation of the peritoneal fluid (Bricou et al., 2008; Hufnagel et al., 2015), disruption of ovarian function, pelvic adhesions, and reduced frequency of sexual intercourse due to dyspareunia (Raffi et al., 2012). Crucially, the infertility associated with OMA may be linked to the negative effect of surgical excision on the ovarian reserve (Raffi et al., 2012; Streuli et al., 2012; Muzii et al., 2007) rather than the OMA lesions themselves (Santulli et al., 2016; Ferrero et al., 2015). Despite decades of research, the pathogenesis of endometriosis remains controversial and poorly understood. Although various factors contribute to its etiology, genetic predisposition accounts for only approximately 7% of cases (Chapron et al., 2010). Furthermore, the combination of several factors including the non-specific nature of the symptoms and inadequate public awareness contributes to a substantial diagnostic delay (Chapron et al., 2003). As a result, the typical delay period between the onset of symptoms and diagnosis has been estimated to be as long as 8 years, underlying the need for new biomarkers allowing an early and accurate diagnosis.

In recent years, microRNAs (miRNAs or miRs) have emerged as powerful post-transcriptional regulators in disease pathogenesis. MiRNAs are small, endogenous non-coding RNA molecules (18–25 nucleotides) that regulate gene expression by binding to target messenger RNAs (mRNAs), leading to the repression of protein synthesis or degradation of the targeted mRNA (Hill and Tran, 2021). A significant body of research has demonstrated a dysregulated miRNA profile in the eutopic and ectopic endometrium of women with endometriosis (Marquardt et al., 2023; Bendifallah et al., 2023), suggesting their critical involvement in altered endometrial biology, the inflammatory cascade, and the fibrotic changes characteristic of the disease. Specifically, miRNAs are implicated in regulating key pathways that contribute to the disease, such as impaired steroid biosynthesis, increased invasive potential associated with neoangiogenesis, and the proinflammatory profile observed in endometrial tissue (Bendifallah et al., 2023). Evidence further suggests that miRNAs can be released from damaged or apoptotic cells (Jiao et al., 2021), potentially constituting a specific circulating miRNA signature reflective of the disease burden. However, their precise role as gene drivers in the overall pathogenesis is still being investigated.

To date, most studies investigating miRNA-based biomarkers for endometriosis have focused on blood-derived samples, including plasma and serum (Vanhie et al., 2019; Moustafa et al., 2020). These studies have identified several candidate miRNAs associated with disease presence and severity, highlighting the potential of circulating miRNAs as non-invasive diagnostic tools (Vanhie et al., 2024; Suryawanshi et al., 2013; Wang et al., 2013; Cho et al., 2015). However, blood-based miRNA analysis presents some limitations, including the invasive nature of sample collection, variability associated with menstrual cycle phase, systemic inflammatory conditions, haemolysis, and other physiological factors that may influence circulating miRNA profiles. These limitations have contributed to the ongoing search for alternative biological matrices suitable for biomarker discovery. Among these, saliva has emerged as an attractive source of diagnostic biomarkers. Saliva collection is simple, safe, non-invasive, and readily repeatable, making it particularly suitable for longitudinal monitoring. In addition, salivary miRNAs exhibit remarkable stability because they are protected within extracellular vesicles or associated with RNA-binding proteins. Accumulating evidence further suggests that salivary miRNAs can reflect systemic physiological and pathological processes rather than exclusively local oral conditions, supporting their use as biomarkers for diseases affecting distant organs.

Based on these considerations, in the present study we investigated the miRNAome in non-invasive samples, represented by saliva obtained by healthy donors (without any gynecological issues) and compared it with that of patients with a confirmed diagnosis of endometriotic lesions or diagnosed with benign gynecological conditions, such adenomas or myomas with the final aim of finding a panel of miRNA with a non-invasive diagnostic potential. We also examined the possible modulation of the miRNAome as a consequence of a therapeutic intervention. Once the cluster with diagnostic potential was identified, we further investigated the biological role of the miRNA forming the cluster in order to understand its biological significance and specificity in the context of endometriosis, in the future perspective of facilitating the diagnosis of such debilitating condition affecting women worldwide.

## 2 Materials and methods

### 2.1 Ethic statement

All procedures in this study were conducted in accordance with the Helsinki Declaration. The study was approved by the Regional Ethics Committee for Clinical Trials of the Region of Tuscany Area Vasta Sud EST (ID23576), by Lombardy Regional Ethics Committee 3 (ID 4165_S_N). The study has also been submitted and registered on ClinicalTrials.gov (ID protocol number: NCT06100471 registration date 10-13-2023). All women enrolled in the study were properly informed and signed an informed consent.

### 2.2 Study cohort and sample collection

**FIGURE 1:**
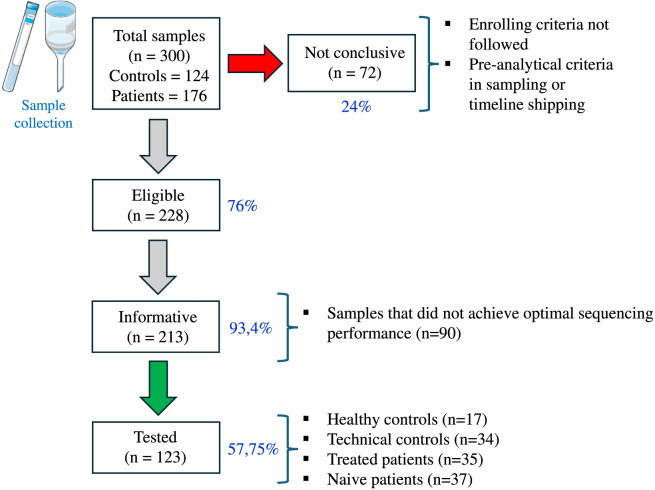
Flow diagram of the study selection process. After the first screening, 72/300 samples were immediately excluded, with 228 considered eligible, and a final inclusion of 213 (informative) salivary samples. Among the informative samples, only 123 were tested, achieving optimal sequencing performance.

The case-control study was conducted considering two patients’ cohorts composed by300 patients. The case group consisted of 176 patients diagnosed with endometriosis, while the control group included a total of 124 subjects belonging to two different sub-groups: healthy (no symptoms) or patients with other gynecological conditions (Figure 1).

Endometriosis was diagnosed either clinically, supported by imaging techniques, or through histological confirmation following surgical intervention. Among 300 collected samples, 228/300 (76%) were eligible for miRNA expression profiling analysis, while 72/300 (24%) were classified inconclusive for eligibility criteria, or not suitable considering pre-analytical criteria in sampling or timeline shipping. Saliva samples were collected by using OMNIgene kit (Danagen-Bioted, S.L., Badalona, Barcellona-Spain) following the manufacturer’s instructions. Salivary sample were obtained by subjects avoiding eating, drinking, or smoking at least 30 min before collection, stored at 4 °C, and shipped within a maximum of 5 days.

In order to improve the interpretability of biomarker data also reducing potential confounding factors, endometriosis cases were further stratified based on their therapeutic status at the time of sample collection. Specifically, patients were further categorized into:

- Treated cases: patients with a diagnosis of endometriosis undergoing pharmacological therapy (e.g., hormonal treatment) or who had recently undergone surgical intervention.
- Untreated cases: patients with a first diagnosis of endometriosis not receiving at the time of the diagnosis any pharmacological treatment or surgical procedures.

The control group was also stratified as follow:

- Technical controls: patients with benign gynecological conditions (e.g., ovarian cysts, fibroids) but without a diagnosis of endometriosis.
- Healthy controls: patients with no gynecological issues, referring to a visit as yearly routine control.

### 2.3 miRNA extraction and quality control

Saliva samples were immediately processed or stored at 4 °C until use. All processes were conducted in an RNase-free environment. Both RNA extraction and miRNA-enrichment were performed by using 1 mL of salivary sample with the miRNeasy Mini Kit and MyQIAcube, (Qiagen, Germantown, MD, USA) according to the manufacturer’s instructions. Briefly, 1 mL of saliva was centrifuged at 3000 RCF for 20 min, the supernatant was removed, and the pellet was resuspended in 700 µL of QIAzol Lysis Reagent (Qiagen). Then, 140 µL of chloroform (Sigma-Aldrich Chemie GmbH, Taufkirchen, Germany) were added followed by a centrifugation at 1200 RCF for 15 min. The supernatants were then collected and used for the subsequent purification of the miRNA-enriched fractions separated from the larger RNAs, by using the miRNeasy Mini Kit and MyQIAcube instrument, according to the manufacturer’s instructions.

### 2.4 Library preparation and next generation sequencing (NGS)

In order to assure performance in downstream applications, following purification, miRNA underwent to an internal Quality Check by using a Qubit Fluorometer (ThermoFisher Scientific, Waltham, MA, USA), with samples having a concentration lower than 15 ng/μL being defined as not compliant for the sequencing analysis. An equal quantity of miRNA (100 ng) was used for each sample analyzed. Compliant miRNAs were subjected to library preparation before sequencing (QIAseq miRNA Library Kit, Qiagen). Sequencing metrics were: 72 bp read lenght and sequencing depth of 10 milion reads, single end. The analysis was performed by using a NextSeq 550 Sequencer with NextSeq 500/550 v2.5 Kits (Illumina, San Diego, CA, USA).

### 2.5 Raw data preprocessing and quality controls

Demultiplexing was performed using the bcl2fastq (v2.20.0.422) software (Illumina), and FASTQ files’ quality was visually inspected using FastQC (v0.11.8) (Andrews, 2010). Reads were filtered using UMI-tools (http://umi-tools.rtfd.io/) and SeqIO python package (https://pypi.org/project/seqio/), to exclude sequences shorter than 16 bp or with missing or incomplete UMI (UMI sequence shorter than 10 bp). Alignment was performed via the bowtie2 software (v2.3.4.1) using miRBase (v22.1) as a database, permitting 1 mismatch and keeping only the best alignment. Eventually, the count matrix was obtained by collapsing reads with UMI tools (with--method cluster). Only samples with at least 10000 reads mapping to the miRNAome were considered for further analysis.

### 2.6 Statistical analysis

A two-steps statistical analysis was carried out, with a first explorative differential expression analysis and a more focused cluster analysis.

#### 2.6.1 Differential expression analysis of miRNAs

The expression level of miRNAs was first estimated using UMI tools, and then differential expression (DE) was performed using R (3.6.3). Samples were labeled based on their clinical classification (Supplementary Table S1), and case group was tested against the control group. The DE model only considered miRNA expression level as the dependent variable, with no additional covariate. Results of the DE were obtained by using the DESeq2 package (v1.26.0) (Love et al., 2014). Data normalization was performed by using DESeq2. Receiver operating characteristic (ROC) curve analysis was performed to assess the ability of the Composite Risk Index (CRI) to discriminate between cases and controls. Diagnostic performance was evaluated by calculating the area under the ROC curve (AUC) with corresponding sensitivity and specificity values. The optimal cutoff value was determined using the maximum Youden index (Supplementary Figure S1).

#### 2.6.2 miRNA prioritization strategy

Candidate miRNAs were selected through a multi-step prioritization process integrating both experimental and literature-derived evidence. First, differential expression analysis was performed on the entire salivary miRNA dataset using DESeq2. Subsequently, miRNAs were filtered according to statistical significance (p-value < 0.05) and sufficient expression abundance across the cohort (average read count >10). The resulting candidate list was then compared with previously published studies investigating miRNA dysregulation in endometriosis. Particular attention was given to miRNAs involved in biological processes known to contribute to endometriosis pathogenesis, including inflammation, angiogenesis, epithelial–mesenchymal transition (EMT), fibrosis, cellular proliferation, and hormone-responsive signalling pathways.

The final panel was selected by integrating statistical significance, expression abundance, consistency with previous literature, and biological relevance based on experimentally validated target genes and pathway analyses. This approach aimed to identify a biologically coherent miRNA signature rather than individual biomarkers showing isolated differential expression.

#### 2.6.3 miRNA cluster analysis

As results from the DE a cluster of 10 miRNAs was selected and evaluated against published data. The representativity of this cluster has been evaluated on the samples that previously underwent DE analysis by considering the share of sample reads which mapped to any of the miRNAs in the cluster, producing a cluster representativity index CRI for the sample. We used finer classification labels in this cluster analysis. Samples were divided based on the classification criteria described in Section 2.2 and the role of symptoms and treatment in miRNAome expression was investigated. For each of the groups, the median value of the CRIs was evaluated (Supplementary Material 1), showing a first clue of clustering behavior.

### 2.7 Gene ontology analyses

Gene ontology (GO) analyses were performed on the genes targeted by miRNAs. Genes targeted by the single miRNAs in the cluster were extracted using miRTarBase (Cui et al., 2025). GO were performed on the genes targeted by at least 5 and at least 4 miRNA using g: Profiler (Raudvere et al., 2019) version e113_eg59_p19_6be52918, accessed on January 24th, 2026. GO analysis regarded biological processes, molecular functions, and cellular components (GO:BP, GO:MF, GO:CC). Statistical domain scope was set to “Annotaded genes only” and significance threshold was set to Set Counts and Sizes (g:SCS threshold) with a threshold at 0.05.

### 2.8 Informed consent

Written informed consent was obtained from all participants, including consent for the publication of anonymized data.

## 3 Results

The first aim of the present study was to identify a non-invasive biomarker panel. A specific cluster of 10 salivary miRNAs, which are highly relevant to the pathophysiology of endometriosis and/or its associated processes, such as inflammation or precancerous lesions, was identified by literature review and differential expression analysis. In terms of the statistics, 7 of the 10 miRNAs (hsa-miR-130a-3p, hsa-miR-130b-3p, hsa-miR-141-3p, hsa-miR-203b-5p, hsa-miR-203a-3p, hsa-miR-29c-3p, hsa-miR-6809-5p) are part of a larger set of 86 miRNAs (47 upregulated, 39 downregulated) showing both significant differences in expression (p-value < 0.05) and a widespread level of expression across the cohort (average of reads > 10). Complete results of the Differential Expression procedure are available at Supplementary Material 1, where the relevant miRNAs are highlighted. In literature, 7 of the 10 miRNAs, namely hsa-miR-130a-3p, hsa-miR-130b-3p, hsa-miR-141-3p, hsa-miR-200b-3p, hsa-miR-200c-3p, hsa-miR-203b-5p, and hsa-miR-29c-3p, are known for their direct correlation with endometriosis, while remaining 3 miRNAs exhibit a clear association with inflammation and/or cancer progression (Table 1).

**TABLE 1:** Cluster of salivary miRNAs correlated to the pathophysiology of endometriosis and/or its associated processes.

miRNA	Main correlation	Molecular targets	References
hsa-miR-6809-5p	Inflammation and Cancer progression	*FLOT1*	Yang et al. (2025)
hsa-miR-200b-3p	Endometriosis	*ZEB1, ZEB2, DMD, NFASC, SNAP25, HDAC4*	Ohlsson Teague et al. (2009), Zhang et al. (2021), Lyu et al. (2026)
hsa-miR-130a-3p	Endometriosis	*SNAP25*	Filigheddu et al. (2010), Baxter et al. (2025)
hsa-miR-130b-3p	Endometriosis	*SNAP25, SPIN90*	Filigheddu et al. (2010), Ahn et al. (2022), Carlino et al. (2018)
hsa-miR-29c-3p	Endometriosis	*CDK6, BCCIP, TCF7L1, TCF7L2, PTEN, COL4A1, E‐Cadherin,* and *N‐Cadherin*	Wentges et al. (2025), Yadav et al. (2026)
hsa-miR-203b-5p	Endometriosis	*VEGFA*	Wang et al. (2022)
hsa-miR-141-3p	Endometriosis	*TGFβ2, ZEB1, ZEB2, PPP1R12A* and *PPP1R12B*	Ohlsson Teague et al. (2009), Yang et al. (2021), Jalouli et al. (2026)
hsa-miR-200c-3p	Endometriosis	*NFASC, SNAP25, MALAT1*	Filigheddu et al. (2010), Liang et al. (2017), Senturk et al. (2026), Xia et al. (2026)
hsa-miR-27a-3p	Inflammation	*IGF1*	Di Pietro et al. (2018)
hsa-miR-203a-3p	Cancer progression	*SLUG, SOCS3/JNK/c-Jun, JAK1, STAT1*	Pashaei et al. (2022), Zhang et al. (2020), kumar et al. (2025)

**FIGURE 2:**
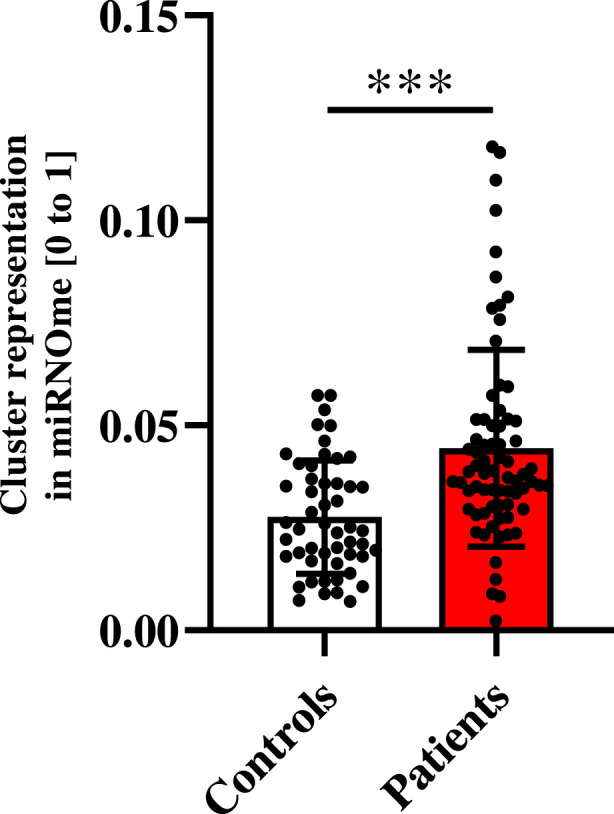
Histograms illustrating the DE of the 10-miRNA cluster in the saliva of controls (n = 51) and patients (n = 72). The Y-axis shows the cluster representation within the miRNome (normalized between 0 and 1). Each dot represents a single patient sample. ***Significantly different, p < 0.001.

Given the hypothesis that endometriosis shares inflammatory and proliferative pathways with cancer, the presence of miRNAs such as hsa-miR-203b-5p (associated with inflammation) and hsa-miR-200c-3p (associated with cancer progression) in the cluster reinforces the biological validity of the panel as a potential disease signature. For classification purposes, the expression value of the cluster was normalized against the total miRNA reads per sample. As reported in Figure 2, a significant difference was observed when comparing the mean cluster expression values between patients with a first endometriosis diagnosis or under clinical management and controls represented by healthy subjects or patients with no diagnosis of endometriosis, but with other gynecological issues.

The analysis of the 10-miRNA cluster had a 1.6-fold increase compared to the control group. We evaluated the discriminative ability of this index by assuming its use as a classification score. Under this assumption, the resulting classifier would achieve an AUC of 0.696 (Supplementary Figure S1). Selecting the optimal threshold (0.0273), the corresponding sensitivity and specificity would be 0.8533 and 0.5273, respectively.

**FIGURE 3:**
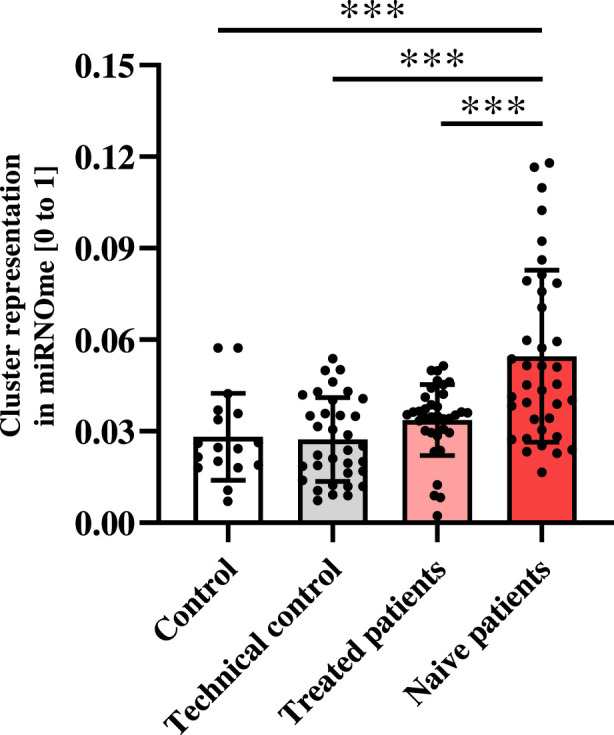
Histrograms illustrating the DE of the 10-miRNA cluster in the saliva of healthy controls (n = 17), technical controls (n = 34), treated patients (n = 35), and naive patients (n = 37). The Y-axis shows the cluster representation within the miRNome (normalized between 0 and 1). Each dot represents a single patient sample. ***Significantly different, p < 0.001.

Despite saliva is recognized as a reliable sample matrix in bioanalytics (Gröschl, 2017), its complexity is intrinsically associated with high inter-individual variability and significant biological noise (Resz et al., 2025), reason why a more rigorous and detailed clinical stratification analysis was performed. This methodological approach had the crucial dual objective of isolating the specific impact of the underlying pathology from the effect due to ongoing drug therapies, while simultaneously validating the robustness of the observed differential miRNA cluster expression pattern. To achieve this finer analysis, the two cohorts, controls and patients, were subsequently disaggregated into clinically more homogeneous sub-clusters represented by healthy controls (patients without symptoms or history of endometriosis), technical controls (patients with no diagnosis of endometriosis, but with other gynecological issues), treated patients (undergoing therapy or recent surgery), and naive patients (first endometriosis diagnosis). The results from this stratification (Figure 3) strongly confirmed the initial finding, highlighting a coherent and gradual progression of the mean miRNA cluster representation going from healthy subjects to naive patients, enlarging the perspective for this cluster to become not only a promising diagnostic tool but also a longitudinal instrument for assessing the efficacy of endometriosis treatment.

As clearly depicted in Figure 3, no significant differences were observed when comparing healthy controls (0.027) to technical controls (0.028), having almost completely overlapping values. Of note, the treated group showed a mean value (0.033) that was similar to that of both control groups, but significantly lower (p < 0.001) compared to that of naive patients (0.054), showing the efficacy of the treatment (therapy or surgery) in negatively modulating the considered 10-miRNA cluster.

**FIGURE 4:**
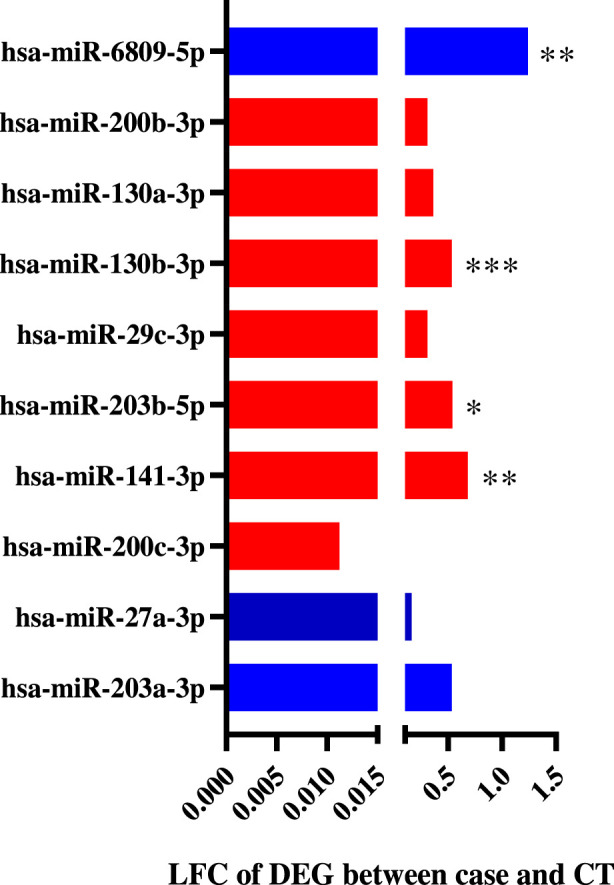
Differential gene expression of the 10 miRNAs in the cluster. Bar graph shows the log fold change (LFC) of differential miRNA expression (DEG) between case and controls for the 10 miRNAs included in the cluster. miRNAs, that are known for their direct correlation with endometriosis are highlighted in red. *Significantly different, p < 0.05; **Significantly different, p < 0.01; ***Significantly different, p < 0.001.

While the increase in the cluster representation index for the cluster of genes in cases compared to the controls is clear, the different contribution of the differential expression of the single miRNAs was worth exploring. The log fold change (LFC) of differential gene expression for the single miRNAs in the cluster is shown in Figure 4.

Among the miRNAs in the cluster, hsa-miR-6809-5p has the biggest differential gene expression of the 10 miRNAs in the cluster between case an CT with a LFC of 1.24 (significant with a p-val<0.01). Other miRNAs have shown a significant differential gene expression: hsa-miR-130b-3p with a LFC of 0.53 (p-val<0.001); hsa-miR-203b-5p with a LFC of 0.54 (p-val<0.05); hsa-miR-141-3p with a LFC of 0.68 (p-val<0.01). The other miRNAs in the cluster have not shown a significant differential gene expression but all of them show an increasing trend in the endometriosis group compared to controls.

**FIGURE 5:**
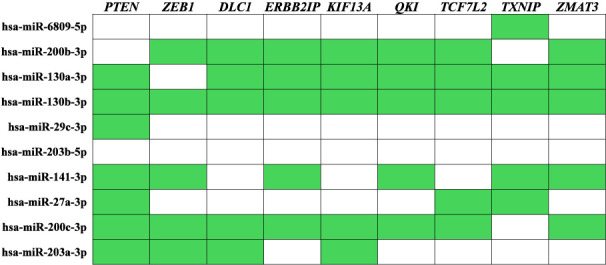
Correspondence miRNA-target for the targets of at least 5 miRNAs. The relationship between the 10 miRNAs in the cluster and their target is reported. Each green square in the table indicates that the miRNA (in y axis) targets the gene (in x axis). Only genes that are targeted by at least 5 miRNA (50%) of the cluster are shown in the cross table of the figure.

To improve the understanding of the role of the 10 miRNAs in the cluster and their involvement in the pathology of endometriosis, genes that are their target were extracted using miRTarBase, an annotated database based on experimentally validated miRNA-target interactions. Target genes were then ranked according to the number of miRNAs they interacted with. The genes that were common target of at least 5 miRNAs are shown in Figure 5, together with the miRNAs they are targeted by.

**FIGURE 6:**
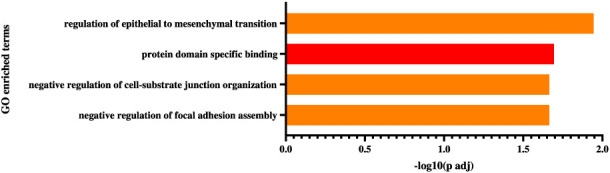
Bar graph showing enriched terms at the gene ontology analysis of the 9 genes that are a common target for at least 5 miRNAs of the 10 miRNAs in the cluster. Most enriched terms are related to regulation of epithelial to mesenchymal transition and negative regulation of cell adhesion.

Interestingly, the Phosphatase and Tensin homolog (*PTEN*) is the most central gene, targeted by 7/10 miRNAs. *PTEN* is an oncosuppresor gene, which targets the mTOR/PI3K/AKT pathway and that is downregulated in tumours (Maphutha et al., 2024). Moreover, what is worth mentioning is that the miRNA hsa-miR203b-5p, which has shown a significant differential gene expression (Figure 4) does not share any of the most common target with other miRNAs, this allowing to hypothesize a different pattern of regulations it is responsible for. On the other hand, hsa-miR-200c-3p, which shows the slightest differential gene expression (Figure 4) with a LFC of 0.01, is very central and shares almost all the most common targets with other miRNAs, suggesting a role in regulating converging pathways altered in the pathology. Given the inhibitory role of miRNAs in eucaryotic organisms, all the genes targeted by the increased expressed miRNA have to be considered downregulated in the pathology (Gebert and MacRae, 2019). The biological processes and molecular functions altered in relationship to the 9 genes that are common target of at least 5 miRNAs was then investigated, and results are shown in Figure 6.

**FIGURE 7:**
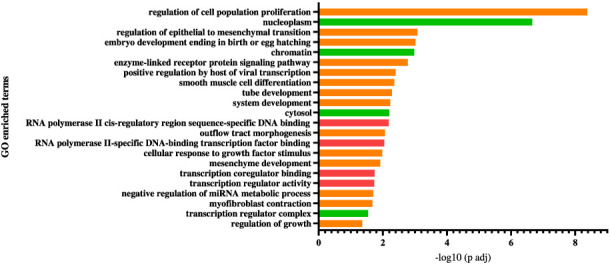
Bar graph showing enriched terms at the gene ontology analysis of the 34 genes that are a common target for at least 4 miRNAs of the 10 miRNAs in the cluster. Enriched terms that appear new in this broader analysis regard regulation of transcription with a focus on RNA polII regulation and embryonal development.

Two main processes emerged from the GO analysis performed, both known to be involved in endometriosis: regulation of epithelial to mesenchymal transition (EMT) (Hosseinirad et al., 2025) and the negative regulation of cell-substrate junctions and focal adhesion (Choi et al., 2018). This shows that the miRNAs emerged from the cluster analysis performed on the miRNAome are targeting pathways strongly related to the pathology. To broaden the understanding of the regulatory network altered by the genes in the cluster, the GO analysis was extended to genes that were target of at least 4 miRNAs, this extending the analysis from 9 to 34 genes, for which the GO is shown in Figure 7. Among these, *JUN*, *SMAD4*, *VEGFA* are some of the most known.

In this case, together with already present EMT regulation, biological processes related to embryonal development (e.g., embryo development ending in birth or egg hatching, tube development, outflow tract morphogenesis) and molecular functions related to the regulation of transcription (e.g., RNA polymerase II cis-regulatory region sequence-specific DNA binding transcription coregulator binding) emerge as significantly relevant, opening new path to study the pathophysiology of endometriosis. The results together give strong molecular support to the relevance of the cluster identified which deserves further studies as a non-invasive tool for the diagnosis of endometriosis pathology.

## 4 Discussion

### 4.1 Salivary miRNAs as non-invasive biomarkers of endometriosis

Endometriosis represents a common chronic gynecological disorder affecting approximately 5%–10% of women of reproductive age; notably, its prevalence may rise to nearly 50% in selected populations, largely due to substantial underdiagnosis. This diagnostic gap is partly due to the absence of reliable, non-invasive diagnostic tools. Currently, the diagnostic “gold standard” for endometriosis remains an invasive laparoscopic surgical procedure often associated to risks of infections and bleeding typically performed in an advanced stage of the pathology (Dunselman et al., 2014; Taylor et al., 2018). Consequently, there is a critical need for accurate, accessible, and non-invasive diagnostic approaches that would enable earlier detection, facilitate timely initiation of medical therapy, and allow longitudinal monitoring of disease recurrence without reliance on repeated laparoscopic interventions. In this context, several efforts have been directed toward the development of non-invasive diagnostic strategies for endometriosis (Avery et al., 2024; Sadeghzadeh Oskouei et al., 2025), and among these the analysis of miRNA expression represents a promising approach for detecting molecular changes associated with pathogenesis and progression of several diseases, including endometriosis (Bjorkman and Taylor, 2019). MiRNAs are present both intracellularly and in circulation and have been detected in multiple body fluids with remarkable stability (Weber et al., 2010; Fehlmann et al., 2016).

Several studies have proved the role of miRNA as potential biomarkers in the plasma of endometriosis patients, allowing to identify multi-marker panels strictly associated to the progression of this disease (Vanhie et al., 2024; Zafari et al., 2022; Rekker et al., 2015; Papari et al., 2020). Numerous miRNAs, including members of the miR-200 family and miR-17-5p, have shown strong correlations with laparoscopic biopsy-derived findings, while additional candidates such as miR-451a, let-7b, miR-20a-5p, and miR-3613-5p have been reported in serum (Zafari et al., 2022; Rekker et al., 2015; Papari et al., 2020; Nothnick et al., 2017; Yu et al., 2025). However, despite the consistency of these results, the diagnostic value of miRNAs remains limited as a consequence of the variability related to the menstrual phase during which samples are collected that may influence miRNA expression, or possible alterations of miRNA levels during proliferative and secretory phases (Hsu et al., 2014; Petracco et al., 2019; Leonova et al., 2021). In this context, saliva-based miRNA profiling represents an attractive and, more importantly, non-invasive alternative, offering easy sample collection and potentially reduced sensitivity to systemic hormonal fluctuations. Despite the high endogenous RNase activity of saliva, salivary miRNAs have been shown to exhibit remarkable stability and reproducibility as molecular biomarkers (Michael et al., 2010; Gallo et al., 2012; Gai et al., 2018; Setti et al., 2020; Faur et al., 2021; Hashm et al., 2026). This stability is primarily attributable to their encapsulation within extracellular vesicles, including exosomes and microvesicles, as well as their association with RNA-binding proteins such as Argonaute complexes, which protect miRNAs from enzymatic degradation. As a result, salivary miRNAs remain detectable under physiological conditions and can reliably reflect molecular alterations occurring in distant tissues. Indeed, salivary miRNA signatures have been successfully investigated as non-invasive biomarkers in a variety of pathological conditions, including cancer, inflammatory disorders, neurodegenerative diseases, and gynaecological diseases (Michael et al., 2010; Kaczor-Urbanowicz et al., 2017). Together with the ease, safety, and non-invasive nature of sample collection, these characteristics support the use of saliva as a reliable biological matrix for assessing miRNA expression patterns associated with endometriosis pathogenesis and related inflammatory and cancer-associated pathways (Bendifallah et al., 2025; Bendifallah et al., 2022; Dabi et al., 2023; Bendifallah et al., 2024; Lyu et al., 2026).

### 4.2 Biological relevance of the identified miRNA signature

In our work we identified a cluster of ten salivary miRNAs strongly associated with endometriosis-related pathways (Table 1). Specifically, seven miRNAs (hsa-miR-130a-3p, hsa-miR-130b-3p, hsa-miR-141-3p, hsa-miR-200b-3p, hsa-miR-200c-3p, hsa-miR-203b-5p, and hsa-miR-29c-3p) have been linked directly to endometriosis, while the remaining three are primarily associated with inflammatory processes and cancer-related pathways. Several miRNAs previously reported in association with endometriosis, including miR-17-5p, miR-451a, and let-7b-5p, were evaluated during the literature review process. However, these candidates were not retained in the final panel because they did not meet all predefined prioritization criteria within the present salivary NGS dataset. To date, only a limited number of studies have investigated salivary miRNAs in endometriosis; for instance, an untargeted high-throughput analysis identified a 109-miRNA salivary signature that lacked independent validation (Bendifallah et al., 2022). In contrast, targeted approaches have identified hsa-miR-135a as significantly upregulated in both saliva and plasma of women with endometriosis (Perricos et al., 2022), supporting its systemic relevance. Consistent with these findings, the inclusion of inflammation- and cancer-associated miRNAs supports the biological relevance of the identified cluster as an endometriosis-associated molecular signature. After normalization, the 10-miRNA cluster showed a significant 1.6-fold increase in patients with endometriosis compared with controls (Figure 2), aligning with mechanistic evidence that key miRNAs implicated in endometriosis, such as hsa-miR-135a, directly target HOXA10 and contribute to impaired endometrial receptivity, dysregulated hormonal signaling, and ectopic lesion development (Petracco et al., 2011). To further validate our findings, the cohort was stratified into healthy controls, technical controls, treated patients, and treatment-naive patients, revealing a progressive increase in miRNA cluster expression from healthy subjects to naive endometriosis patients. Notably, treated patients showed expression levels comparable to controls and significantly lower than naive patients, indicating that medical or surgical treatment effectively modulates this miRNA signature (Figure 3). We investigated the specific contribution of each miRNA belonging to the identified cluster associated with endometriosis (Figure 4) and despite the evident overall increase of the miRNA cluster in endometriosis patients, analysis of individual components revealed heterogeneous contributions. hsa-miR-6809-5p showed the strongest differential expression between cases and controls, followed by significant upregulation of hsa-miR-130b-3p, hsa-miR-203b-5p, and hsa-miR-141-3p. While the remaining miRNAs did not reach statistical significance individually, all exhibited a consistent upward trend in endometriosis patients, supporting the cumulative discriminatory power of the cluster. Our results are in line with the previous data described by Filigheddu et al., in which our salivary 10-miRNA cluster overlap with differential miRNA depicted in both eutopic and ectopic endometrial tissues derived from women affected by endometriosis (Filigheddu et al., 2010). In particular, it has been described a dysregulation of members of the miR-200 family, including hsa-miR-200c-3p and hsa-miR-141-3p, in ectopic lesions strictly correlated with pathways involved in epithelial–mesenchymal transition (EMT), cell proliferation, and hormonal signaling. The overlap of tissue and salivary miRNAs suggests that salivary profiles mirror tissue-level molecular alterations in endometriosis, supporting their value as non-invasive biomarkers.

### 4.3 miRNA-target networks and pathogenic pathways

To better investigate the biological role of the 10-miRNA cluster and, mainly, their involvement in the pathological processes undergone endometriosis, experimentally validated target genes have been identified using miRTarBase and ranked based on the number of interacting miRNAs. *PTEN* emerged as the most central target, being regulated by 7 of the 10 miRNAs (Figure 5), highlighting convergence on the PI3K/AKT/mTOR pathway, which is known to be dysregulated in endometriosis (Madanes et al., 2020). In fact, *PTEN*-mediated regulation of PI3K/AKT signaling is well established in endometrial cancer, where *PTEN* loss drives disease progression by promoting cellular proliferation and resistance to TGFβ-induced apoptosis. In this context, the miR-424 (322)∼503 cluster has been identified as a key regulator of these processes (Vidal-Sabanés et al., 2025).

Included in our 10 miRNAs cluster, hsa-miR-203b-5p has shown a distinct regulatory profile, targeting unique genes not shared with other cluster members, while hsa-miR-200c-3p, even though minimal differential expression, has occupied a central position by sharing most common targets, suggesting a role in coordinating convergent pathogenic pathways (Figure 5).

The specific crosstalk between miRNA dysregulation in endometriosis and other chronic proliferative disorders is represented by the hsa-miR-203b-5p and hsa-miR-200c-3p interconnection. Hsa-miR-200c-3p is a key regulator of EMT and cellular plasticity, processes that are crucial for both tumor invasion and endometriotic lesion establishment. In contrast, hsa-miR-203b-5p is primarily associated with inflammatory signaling and epithelial differentiation, suggesting a complementary role in modulating immune responses and tissue remodeling. The concurrent dysregulation of these miRNAs supports a model in which endometriosis shares cancer-like regulatory networks involving EMT, inflammation, and aberrant cell survival, while maintaining disease-specific molecular features. Our results are corroborated by the study of Góźdź et al. (Góźdź et al., 2025), which demonstrated that EMT-related genes and miRNAs, including miR-200 family, are constitutively expressed in eutopic endometrium and are mostly regulated by the menstrual cycle rather than by disease status, with no evidence of overt EMT activation in eutopic tissue from endometriosis patients. Based on these concepts, the presence of miR-200c-3p within our 10-miRNA cluster suggests that salivary miRNAs more likely reflect systemic or ectopic lesion-derived molecular alterations rather than eutopic endometrial EMT changes, supporting the idea that salivary miRNA profiles reflect signals from ectopic lesions and inflammatory microenvironments, reinforcing, once again, their utility as non-invasive biomarkers tool.

Our findings related to the functional enrichment analysis of genes further support the disruption of key regulatory networks in endometriosis, identifying at least five miRNAs (Figure 7) involved in two major biological processes as EMT and negative regulation of cell-substrate junctions and focal adhesion, both of which exert a central role in the endometriosis pathophysiology. Extending the analysis to genes targeted by multiple miRNAs, we identified SMAD4, together with *JUN* and VEGFA, as central nodes within miRNA–mRNA interaction networks. In fact, in line with our findings, it has been suggested that miR-542-3p-mediated repression of the *BMP7*–*SMAD4*–*CDH1* axis may contribute to the acquisition of mesenchymal-like features in ectopic endometrial lesions. Notably, the concomitant downregulation of *BMP7*, *SMAD4*, and *CDH1* in ectopic tissue and the augmented miR-542-3p expression level suggest that post-transcriptional regulation may play a role in destabilizing epithelial integrity and promoting *EMT*-related processes (Zubrzycka et al., 2023).

Concerning the *JUN* pathway, which is primarily implicated in endometriosis through the AP-1 transcription factor complex, evidence indicates that it plays a key role in promoting invasive behavior. *JUN/AP-1* activity has been associated with the regulation of *EMT*-related genes, adhesion molecules, and extracellular matrix remodelers, and experimental knockdown of *JUN* has been shown to suppress invasion in highly invasive endometriotic cells (Wilson et al., 2022). Emerging evidence suggests that miRNA dysregulation may contribute to altered *JUN/AP-1* signaling in endometriosis. Several disease-associated miRNAs are predicted or validated regulators of *JUN* or its upstream activators, and integrative miRNA–mRNA analyses frequently identify *JUN* as a hub gene within networks linked to inflammation and angiogenesis, supporting a role for post-transcriptional regulation of AP-1-related pathways in disease pathogenesis (Ohlsson Teague et al., 2009; Liu et al., 2022; Li et al., 2025; Burney and Giudice, 2012). Finally, a recent multi-fluid miRNA profiling study further supports the involvement of systemic miRNA dysregulation in endometriosis (Lyu et al., 2026). Distinct miRNA signatures were identified across serum, saliva, and vaginal mucus, yet pathway analyses converged on inflammation, remodeling, angiogenesis, and *TGF-β*-related signaling, emphasizing network-level regulation rather than single biomarkers (Lyu et al., 2026; Bendifallah et al., 2022; Guo et al., 2016; Suárez and Sessa, 2009; Sun et al., 2018; Miscianinov et al., 2018). This is consistent with our identification of a coordinately upregulated salivary miRNA cluster and the convergence of multiple miRNAs on key targets such as *PTEN*, *JUN*, and *SMAD4*, supporting a role for post-transcriptional control of central signaling nodes in endometriosis pathogenesis.

### 4.4 Clinical implications and future perspectives

Collectively, our study identifies a salivary miRNA signature associated with endometriosis, supporting the use of saliva as a feasible and truly non-invasive biological matrix for disease investigation. The coordinated upregulation of a miRNA cluster converging on key signaling nodes, including *PTEN*, *JUN*, and *SMAD4*, highlights the relevance of miRNA-mediated regulatory networks in pathways related to inflammation, angiogenesis, tissue remodeling, and EMT. Notably, our findings suggest that systemic miRNA dysregulation characterizes endometriosis and that salivary miRNAs may reflect underlying pathogenic mechanisms regulated by treatment. However, a possible limitation of the present pilot study is the lack of independent qRT-PCR validation of the 10-miRNA signature identified by NGS. Future studies will validate the expression of the selected salivary miRNAs by qRT-PCR, including direct comparison between healthy controls and treatment-naïve endometriosis patients, strengthening the robustness and reproducibility of our results.

Although further validation is required, these results indicate that miRNA-based signatures detected in saliva could represent promising predictive biomarkers for endometriosis, offering potential clinical utility for early, non-invasive diagnosis and disease monitoring.

## Data Availability

The data presented in the study are deposited in the Figshare repository, available at https://doi.org/10.6084/m9.figshare.32968895.

## Supplementary material

SUPPLEMENTARY FIGURES 1 Receiver operating characteristic (ROC) curve for CRI-based discrimination between cases and controls. The ROC analysis yielded an area under the curve (AUC) of 0.696, indicating moderate discriminatory performance. The optimal cutoff value (CRI = 0.03) was associated with a sensitivity of 85% and a specificity of 53%. The diagonal line represents the performance of a random classifier.

SUPPLEMENTARY TABLES 1 Differential expression analysis of circulating miRNAs in cases versus controls. Differential expression analysis was performed to identify miRNAs significantly altered between cases and controls. For each miRNA, the table reports mean normalized counts, log fold change (LFC), raw p-value, and multiple-testing adjusted p-value (false discovery rate, FDR). Positive LFC values indicate higher expression in cases relative to controls, whereas negative LFC values indicate lower expression in cases. The “DE” column indicates whether the miRNA was classified as differentially expressed according to the predefined statistical significance criteria.
