# Supplementary material for: Profiling miRNA in salivary samples from subjects with endometriosis: a pilot study
Source: Front Mol Biosci. 2026 Jul 22;13:1869937. doi: 10.3389/fmolb.2026.1869937 (PMC13439741; doi:10.3389/fmolb.2026.1869937)
Supplement: Supplementary file 1 [file Image1.pdf]

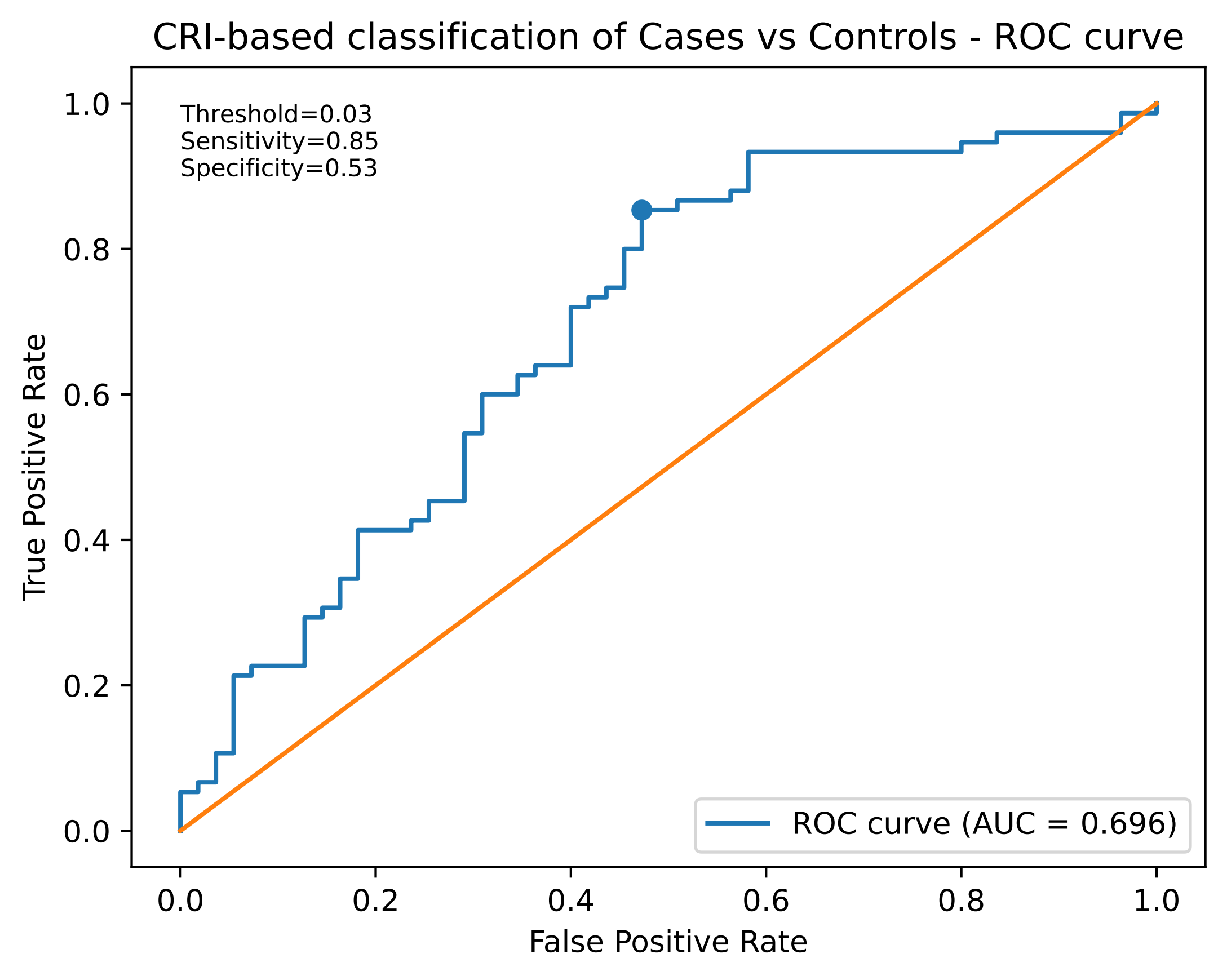
CRI-based classification of Cases vs Controls - ROC curve
Threshold=0.03
Sensitivity=0.85
Specificity=0.53
ROC curve (AUC = 0.696)
True Positive Rate
False Positive Rate
0.0
0.2
0.4
0.6
0.8
1.0
